# Metabolic interaction between amino acid deprivation and cisplatin synergistically reduces phosphoribosyl-pyrophosphate and augments cisplatin cytotoxicity

**DOI:** 10.1038/s41598-020-76958-7

**Published:** 2020-11-16

**Authors:** Nisreen Wahwah, Debanjan Dhar, Hui Chen, Shunhui Zhuang, Adriano Chan, Darren E. Casteel, Hema Kalyanaraman, Renate B. Pilz, Gerry R. Boss

**Affiliations:** Department of Medicine, University of California, San Diego, La Jolla, CA 92093-0652 USA

**Keywords:** Biochemistry, Cancer, Oncology

## Abstract

Cisplatin is a mainstay of cancer chemotherapy. It forms DNA adducts, thereby activating poly(ADP-ribose) polymerases (PARPs) to initiate DNA repair. The PARP substrate NAD^+^ is synthesized from 5-phosphoribose-1-pyrophosphate (PRPP), and we found that treating cells for 6 h with cisplatin reduced intracellular PRPP availability. The decrease in PRPP was likely from (1) increased PRPP consumption, because cisplatin increased protein PARylation and PARP1 shRNA knock-down returned PRPP towards normal, and (2) decreased intracellular phosphate, which down-regulated PRPP synthetase activity. Depriving cells of a single essential amino acid decreased PRPP synthetase activity with a half-life of ~ 8 h, and combining cisplatin and amino acid deprivation synergistically reduced intracellular PRPP. PRPP is a rate-limiting substrate for purine nucleotide synthesis, and cisplatin inhibited de novo purine synthesis and DNA synthesis, with amino acid deprivation augmenting cisplatin’s effects. Amino acid deprivation enhanced cisplatin’s cytotoxicity, increasing cellular apoptosis and DNA strand breaks in vitro*,* and intermittent deprivation of lysine combined with a sub-therapeutic dose of cisplatin inhibited growth of ectopic hepatomas in mice. Augmentation of cisplatin’s biochemical and cytotoxic effects by amino acid deprivation suggest that intermittent deprivation of an essential amino acid could allow dose reduction of cisplatin; this could reduce the drug’s side effects, and allow its use in cisplatin-resistant tumors.

## Introduction

Cisplatin is active against a wide variety of solid tumors, including bladder, head and neck, lung, ovarian, and testicular cancers, and is required for curative treatment of some of these cancers^1^. Its use is limited by toxic side effects and tumor resistance, with both acquired and intrinsic resistance observed^2^.

Cisplatin acts, in part, by covalently binding to the N7 position of adenine and guanine in DNA, generating intra- and inter-strand adducts^1, 3^. In response to such DNA damage, poly(ADP-ribose) polymerases (PARPs) are activated, catalyzing both auto-poly(ADP-ribosyl)ation (PARylation) of a central glutamate-rich domain, as well as PARylation of chromatin-associated proteins. This generates branching polymers of ADP-ribose, which serve as scaffolds for DNA repair factors, including those of the mismatch repair and nucleotide exchange repair pathways^3^. PARPs use NAD^+^ as a substrate, and since NAD^+^ synthesis requires two molecules of 5-phosphoribose-1-pyrophosphate (PRPP), we hypothesized that cisplatin could deplete intracellular PRPP, a rate-limiting substrate for purine, pyrimidine, and pyridine nucleotide synthesis.

We have shown previously that depriving cells of a single essential amino acid decreases PRPP production, due in part to decreased activity of transketolase, a key enzyme in the non-oxidative pentose phosphate pathway^4–6^. Both the oxidative and non-oxidative pentose phosphate pathways generate the PRPP precursor ribose 5-phosphate, with the relative contribution of each pathway varying depending on the cell type and culture conditions^7, 8^. The two main mammalian PRPP synthetases, which convert ribose 5-phosphate to PRPP, are multimers of a single polypeptide chain that aggregate into 4, 8, 16, and 32 subunit complexes, with highest activity in the most aggregated states^9^. Aggregation is regulated, in part, by the intracellular phosphate concentration^9, 10^.

We found that cisplatin decreased intracellular PRPP, and that depriving cisplatin-treated cells of a single essential amino acid profoundly reduced PRPP availability and purine and DNA synthesis. The reduction in PRPP was due largely to decreased PRPP synthetase activity and increased PRPP consumption. The combination of cisplatin and amino acid deprivation reduced tumor cell growth both in vitro and in vivo, with the in vivo effect occurring under conditions where each modality alone had no significant effect.

## Results

### Cisplatin decreases PRPP availability, purine and DNA synthesis, and intracellular ATP and NAD: augmentation by amino acid deprivation

#### PRPP availability

**Figure 1:**
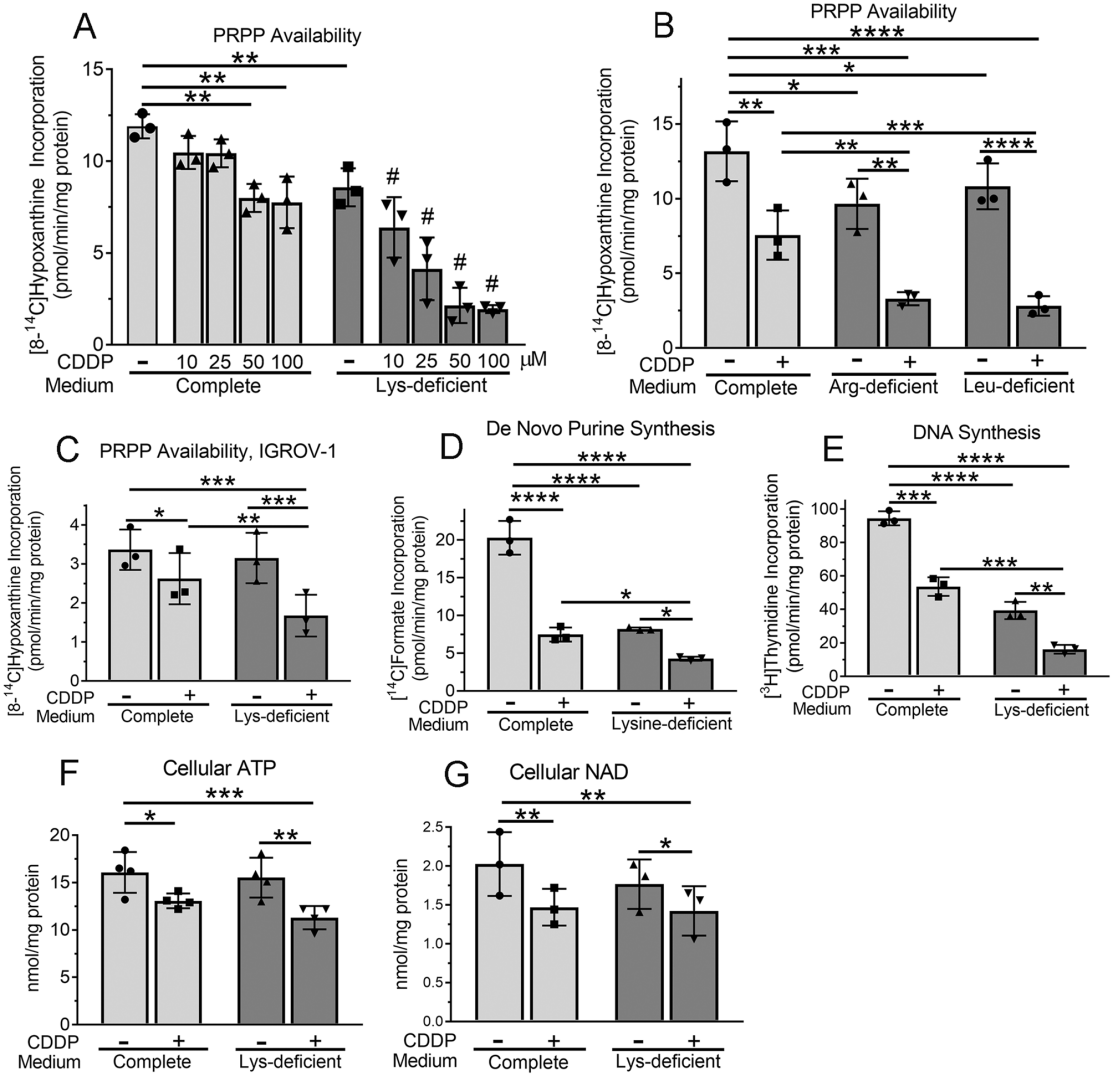
Cisplatin decreases PRPP availability and purine and DNA synthesis, and reduces intracellular ATP and NAD: augmentation by amino acid deprivation. Dih10 cells (**A, B, D**–**G**) or IGROV-1 cells (**C**) were incubated for 6 h (**A**–**D**), 3 h (**E**), or 14 h (**F,G**) in complete medium or lysine-, arginine-, or leucine-deficient medium in the absence or presence of the indicated concentrations of cisplatin (**A**), 100 µM cisplatin (**B,D**–**G**), or 35 µM cisplatin (**C**). (**A**–**C**) [8-^14^C]-hypoxanthine incorporation into purine nucleotides was measured during the last 20 min of the incubation to assess PRPP availability. (**D**) [^14^C]-formate incorporation into purines was measured during the last 90 min of the incubation as a measure of de novo purine synthesis. (**E**) [^3^H]-thymidine incorporation into DNA was measured during the last 60 min of the incubation. (**F,G**) The intracellular content of ATP and NAD were measured by a luciferase-based and a cycling colorimetric assay, respectively. In all panels, each symbol in the bar graphs is the mean of duplicate samples from an independent experiment, with the bar height representing the mean value of all experiments and the error bars indicating the standard deviation. *, **, ***, and **** indicate *p* < 0.05, < 0.01, < 0.001, and < 0.0001, respectively, for the indicated comparisons. # in Panel A indicates *p* < 0.001 for comparison between complete and lysine-deficient medium for each of the four cisplatin concentrations. CDDP, cisplatin; PRPP, phosphoribosyl-pyrophoshate.

The intracellular concentration of PRPP is low and PRPP is relatively unstable, making it difficult to measure PRPP accurately^11^. We, and others, therefore, have measured “PRPP availability” by following incorporation of radioactive hypoxanthine or adenine into purine nucleotides^6, 11–13^. This measures the amount of PRPP available to a cell over a set time interval, as opposed to measuring the PRPP concentration at one time point. We found that treating Dih10 mouse hepatoma cells for 6 h with 10 or 25 µM cisplatin minimally affected PRPP availability, whereas 50 and 100 µM cisplatin reduced PRPP availability by ~ 35% (Fig. 1A, light grey bars; in this figure, and subsequent figures, the control condition is complete medium without cisplatin). The assay is dependent on the enzyme hypoxanthine–guanine phosphoribosyl-transferase (HPRT), and we showed that HPRT activity measured in cell extracts did not change after 6 h of 100 µM cisplatin treatment (Table 1). At the standard human cisplatin dose of 100 mg/m^2^, the cisplatin plasma concentration reaches 30–50 µM^14, 15^; thus, the cisplatin concentrations used were within the range found in patients’ plasma.

**Table 1:** HPRT activity.

Condition	Enzyme activity (nmol/min/mg protein)
Control	1.10 ± 0.19
Lys^−^	1.21 ± 0.17
CDDP	1.14 ± 0.16
Lys^−^/CDDP	1.03 ± 0.18

Dih10 mouse hepatoma cells were incubated for 6 h in full medium (Control), lysine-free medium (Lys^−^), full medium containing 100 µM cisplatin (CDDP), or lysine-free medium containing 100 µM cisplatin (Lys^−^/CDDP). The cells were extracted, and HPRT activity in the extracts was measured following [8-^14^C] hypoxanthine incorporation into nucleotides in the presence of 1 mM PRPP. Data are the mean ± S.D. of three independent experiments.

Depriving Dih10 cells of lysine, arginine, or leucine for 6 h reduced PRPP availability by ~ 25% (Fig. 1A,B). Combining amino acid deprivation with cisplatin strikingly reduced PRPP availability. Augmentation by lysine deprivation occurred at 10, 25, 50, and 100 µM cisplatin, reducing PRPP availability at 50 and 100 µM cisplatin by ~ 85% (Fig. 1A). Arginine and leucine deprivation were studied at 100 µM cisplatin, the standard cisplatin concentration used in most of the remainder of studies in Dih10 cells, and we found that the combination of amino acid deprivation and cisplatin reduced PRPP availability by 79 and 76%, respectively (Fig. 1B). To determine if cisplatin and amino acid deprivation were synergistic in reducing PRPP availability, we calculated the coefficient of drug interaction (CDI), with amino acid deprivation considered as a drug (CDI calculation is described in Experimental Procedures). We found that 100 µM cisplatin combined with lysine, arginine, or leucine deprivation yielded CDI values of 0.3, 0.5, and 0.4, respectively, indicating strong synergy. We could not use the method of Chou and Talalay to calculate the combination index, because this method requires multiple concentrations of both drugs^16^; at the low concentrations of amino acids where we would have to conduct the experiments, the dose response is steep and the amino acid concentration decreases substantially during the experiment, thus leading to major inaccuracy. Amino acid deprivation did not affect HPRT activity over the time course studied (Table 1).

To determine if cisplatin reduced PRPP availability in other cell types, we examined human ovarian cancer cells. We found that 6 h of cisplatin treatment significantly reduced PRPP availability in IGROV-1 cells as well (Fig. 1C; these studies were conducted at 35 µM cisplatin, because IGROV-1 cells are more sensitive to cisplatin than Dih10 cells). Combining lysine deprivation with cisplatin further reduced PRPP availability, yielding a CDI of 0.7, again indicating synergy (Fig. 1C).

#### Purine and DNA synthesis

PRPP is a key regulator of purine nucleotide synthesis, and we found that cisplatin treatment and lysine deprivation each reduced de novo purine synthesis by ~ 60%, and that combined together they reduced purine synthesis by ~ 80% (Fig. 1D). Reduced purine nucleotide synthesis might be expected to reduce DNA synthesis, and we found that cisplatin treatment and lysine deprivation individually reduced DNA synthesis by 43% and 59%, respectively, and that the combination of the two modalities reduced DNA synthesis by 83% (Fig. 1E; data are for a 3 h incubation; at 6 h DNA synthesis was completely halted under the condition of combined cisplatin treatment and lysine deprivation).

#### ATP concentration

Decreased PRPP availability and purine nucleotide synthesis should decrease cellular ATP, and we found that 14 h of cisplatin treatment decreased the intracellular ATP concentration, with lysine deprivation of cisplatin-treated cells causing little additional decrease in ATP (Fig. 1F). The decrease in cellular ATP is noticeably less than the decrease in PRPP availability and purine synthesis, likely because the cellular ATP pool is large and decreased purine synthesis is reflected slowly in the total ATP concentration. Consistent with this notion, we found no change in the intracellular ATP concentration at treatment times of 8 h or less.

#### NAD concentration

PRPP is also required for synthesis of pyridine nucleotides, i.e., NAD and NADP, and 14 h of cisplatin treatment decreased intracellular NAD (Fig. 1G). Depriving cisplatin-treated cells of lysine had no additional effect (Fig. 1G). Again, these changes are noticeably less than the decrease in PRPP, likely because the NAD pool is also relatively large.

### Cisplatin decreases glucose incorporation into DNA and RNA via the non-oxidative pentose phosphate pathway, and reduces PRPP synthetase activity by reducing intracellular phosphate; augmentation by amino acid deprivation

#### Pentose phosphate pathway

**Figure 2:**
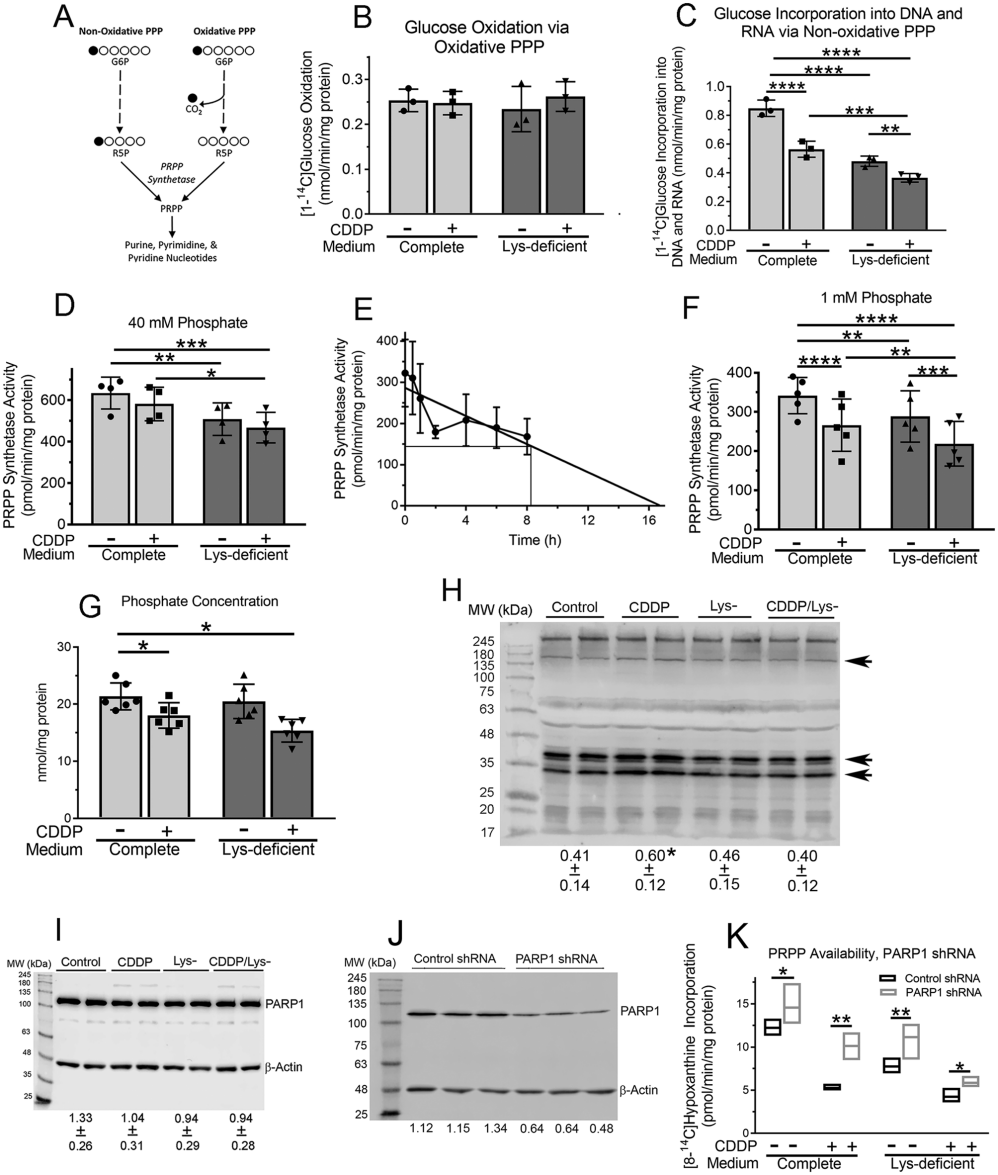
Cisplatin decreases glucose incorporation into DNA and RNA via the non-oxidative pentose phosphate pathway, reduces PRPP synthetase activity by reducing intracellular phosphate, and increases protein PARylation: effects of amino acid deprivation; PARP1 Knock-down Increases PRPP Availability. (**A**) Carbon 1 of glucose (depicted by the filled circle) is oxidized to CO_2_ on glucose transit through the oxidative pentose phosphate pathway, whereas it can be incorporated into PRPP and subsequently into DNA and RNA after transit through the non-oxidative pentose phosphate pathway. (**B-D,F-I,K**) Dih10 cells were incubated for 6 h in complete or lysine-deficient medium in the absence or presence of 100 µM cisplatin. (**B**,**C**) [1-^14^C]-glucose oxidation to CO_2_ (**B**) or incorporation into DNA and RNA (**C**) was measured during the last 60 min of the incubation. (**D**,**F)** Cells were extracted, and PRPP synthetase activity was measured in the extracts in the presence of 40 mM (**D**) or 1 mM (**F**) NaPO4, pH, 7.4 added to the assay buffer. (**E**) Dih10 cells were incubated in lysine-deficient medium, and at the indicated times, PRPP synthetase activity was measured in cell extracts in the presence of 40 mM NaPO4, pH 7.4. (**G**) Inorganic phosphate in cell extracts was measured using a colorimetric assay. (**H**–**J**) Cells were extracted, and proteins were analyzed by SDS-PAGE/immunoblotting. The density of the indicated protein bands was quantified using a Li-COR Odyssey Scanner, and normalized to β-actin. The experiments were repeated three times, with the numbers below the blots showing the mean density ± SD for each of the four conditions (**H**,**I**) or for the indicated lanes (**J**). Full blots are shown. (**H**) An antibody that detects poly(ADP-ribose) polymers was used. The sum of the density of the two bands corresponding to molecular weights of approximately 38 and 33 kDA was quantified; β-actin was imaged on a separate immunoblot. (**I**) A PARP1 antibody was used. (**J**) Clonally-derived Dih10 cells that had been infected with either a control shRNA vector or a PARP1 shRNA vector were extracted, and a PARP1 antibody was used. Each lane corresponds to an independently-derived clone. (**K**) PRPP availability was measured in the three independently-derived clones shown in Panel J, as described in the Fig. 1 legend. Clones infected with control virus are shown in black-outlined boxes, and clones infected with the PARP1 shRNA viral vector are shown in grey-outlined boxes. In Panels B–D, and F and G, each symbol in the bar graphs is the mean of duplicate samples from an independent experiment, with the bar height representing the mean value of all experiments and the error bars indicating the standard deviation. In Panel E, the symbols are the mean of three independent experiments performed in duplicate, and the error bars are the standard deviation. In Panel K, the boxes show the full range of the three independently-derived clones, each measured in duplicate, and the horizontal line in the box is the mean value. *, **, ***, and **** indicate *p* < 0.05, < 0.01, < 0.001, and < 0.0001, respectively, for the indicated comparisons, or in Panel H to the control condition. CDDP, cisplatin; PARP1, poly(ADP-ribose) polymerase-1; PPP, pentose phosphate pathway; PRPP, phosphoribosyl-pyrophosphate.

PRPP can be produced via the oxidative and non-oxidative pentose phosphate pathways. Assessing carbon flow through the oxidative pentose phosphate pathway is generally done by following [1-^14^C]glucose oxidation to [^14^C]carbon dioxide (Fig. 2A), realizing that some oxidation of the C-1 carbon of glucose can occur in the tricarboxylic acid cycle^17^. Using this method, we found no change in oxidative pentose phosphate pathway activity after 6 h of cisplatin treatment, lysine deprivation, or the combination of the two (Fig. 2B). Assessing carbon flow through the non-oxidative pentose phosphate pathway is more difficult, because the pathway is not linear and several reactions are at equilibrium. We have shown previously that following [1-^14^C]glucose incorporation into nucleic acids can be used as a measure of non-oxidative pentose phosphate pathway activity, recognizing this method also assesses PRPP synthetase activity as well as multiple downstream steps (Fig. 2A)^6, 7, 18^. Using this method, we found a marked reduction in glucose incorporation into DNA and RNA after 6 h of either cisplatin treatment or lysine deprivation, with the combination of the two modalities further reducing glucose incorporation (Fig. 2C). The lack of a change in glucose oxidation by cisplatin treatment, lysine deprivation, or the combination of the two indicates that the cause of their reduction in glucose incorporation into nucleic acids occurred distal to glucose uptake and phosphorylation.

#### PRPP synthetase activity and intracellular phosphate

PRPP synthetases are highly regulated and are rate-limiting enzymes for purine nucleotide synthesis^19^. To determine if the enzymes were affected by cisplatin treatment and/or amino acid deprivation, we measured enzyme activity in cell extracts, and found no change in activity in extracts derived from cisplatin-treated cells in the presence of 40 mM phosphate in the assay buffer, a phosphate concentration where the enzymes should be activated maximally (Fig. 2D)^9, 10^. Under these conditions, lysine deprivation significantly reduced enzyme activity, and the combination of cisplatin and lysine-deprivation yielded similar activity as lysine deprivation alone (Fig. 2D). Since lysine deprivation quickly reduces protein synthesis^4^, these data suggest that PRPP synthetase(s) may have a relatively short half-life. Repeated measures of PRPP synthetase activity over 8 h of lysine deprivation yielded a linear decrease in activity, with a 50% loss of activity by ~ 8 h (Fig. 2E).

We next measured PRPP synthetase activity in the presence of 1 mM phosphate in the assay buffer, a phosphate concentration that is limiting to enzyme activity so that phosphate in the cell extracts will contribute to enzyme activity. We now found decreased enzyme activity in cisplatin-treated cells (Fig. 2F). Lysine deprivation decreased enzyme activity to a similar extent as in high phosphate buffer, and the combination of cisplatin and lysine deprivation further decreased enzyme activity (Fig. 2F). These data suggested that cisplatin decreased the intracellular phosphate concentration, which could occur through increased protein PARylation, since each ADP-ribose unit contains two phosphates groups; previous workers have observed increased protein PARylation in cisplatin-treated cells^20, 21^. We, therefore, measured inorganic phosphate in cell extracts and found that cisplatin reduced the phosphate concentration by 16% compared to control cells (Fig. 2G). Although this is a relatively small decrease in phosphate concentration, it could have a major impact on PRPP synthetase activity, due to the enzymes’ high phosphate sensitivity at phosphate concentrations < 10 mM^22^. Lysine deprivation had no effect on intracellular phosphate, and the combination of cisplatin and lysine deprivation yielded a similar decrease in intracellular phosphate as cisplatin alone (Fig. 2G).

### Cisplatin reduces PRPP availability through increased protein PARylation and PRPP consumption; amino acid deprivation prevents the increased protein PARylation

The amount of PRPP available to a cell is determined by the amount of PRPP synthetase activity and the amount of PRPP consumed in PRPP-dependent reactions. NAD synthesis consumes two PRPP molecules, and since NAD is the substrate of PARP, increased protein PARylation could reduce PRPP availability. To assess this possibility, we first confirmed increased protein PARylation in cisplatin-treated Dih10 cells (Fig. 2H; note increased PARylation of proteins at molecular weights of ~ 160, 38, and 33 kDa; the 38 and 33 kDa bands are quantified). The increased protein PARylation occurred in the absence of a significant change in PARP1 expression (Fig. 2I). We then used an shRNA approach to reduce PARP1, which accounts for most cellular PARylation activity^3^, and derived independent clones of cells infected with control virus or the PARP1 shRNA virus. PARP1 protein was reduced an average of 58% in cells infected with the PARP1 shRNA vector, compared to cells infected with control vector (Fig. 2I). In cisplatin-treated cells, PARP1 knock-down increased PRPP availability almost two-fold, to within 80% of that found in cells infected with control vector (Fig. 2J). PARP1 knock-down also increased PRPP availability in cells in control medium, lysine-free medium, and lysine-free medium in the presence of cisplatin, although the increase was less than in cells treated with cisplatin alone (Fig. 2J; PRPP availability increased by 20, 44, and 38% in control, lysine-deprived, and lysine-deprived cells treated with cisplatin, respectively). Of note, protein PARylation was unchanged in lysine-deprived cells, but lysine deprivation prevented the increase in protein PARylation found in cisplatin-treated cells (Fig. 2H). The inhibition of protein PARylation in lysine-deprived cells occurred in the absence of a significant change in PARP1 protein (Fig. 2I). We will consider the implications of these findings in the Discussion.

### Cisplatin cytotoxicity and induction of apoptosis and DNA strand breaks are augmented by amino acid deprivation

**Figure 3:**
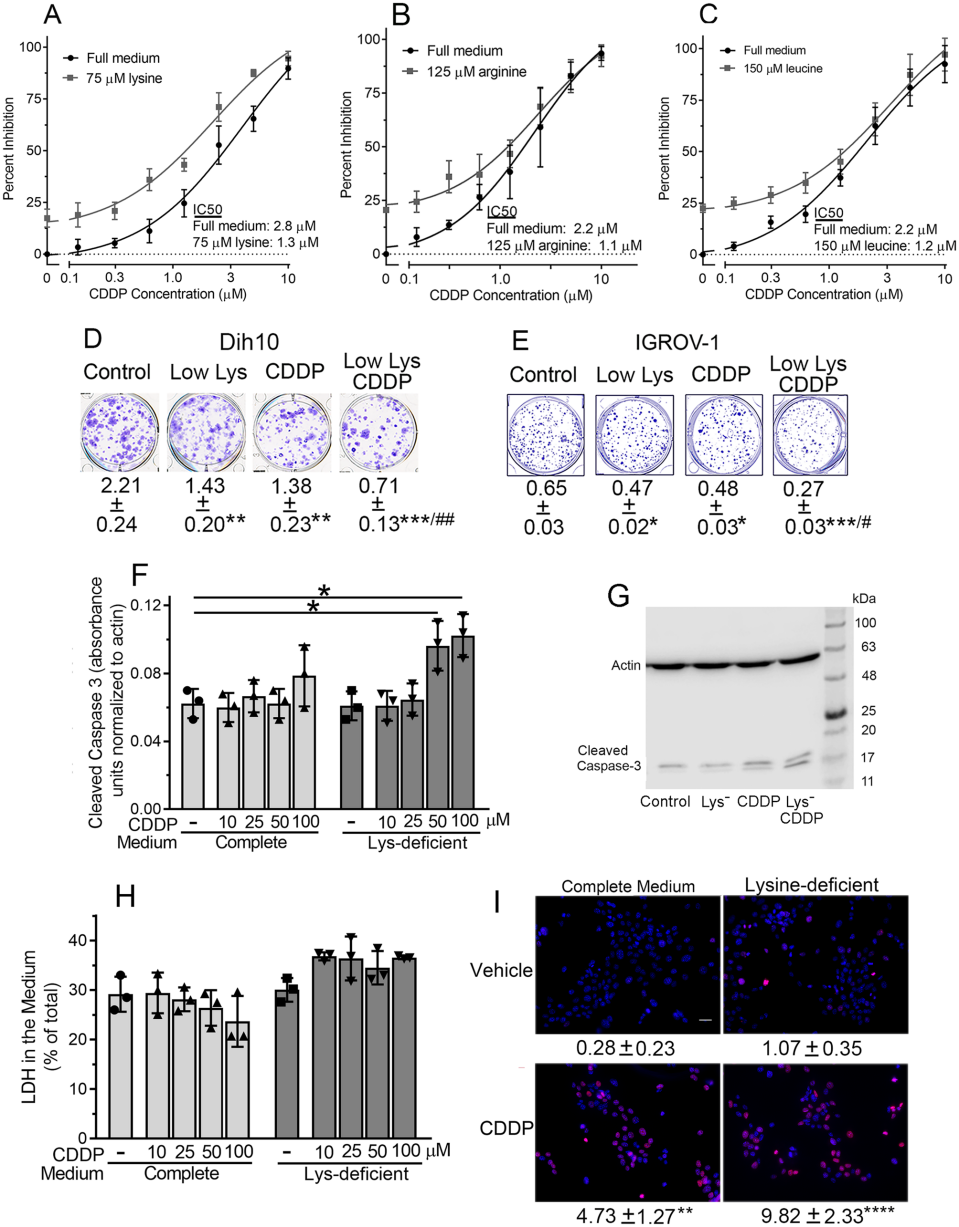
Cisplatin inhibition of cell growth and increase of apoptosis and DNA strand breaks is augmented by amino acid deprivation. (**A**–**C)** Dih10 cells were cultured in the presence of the indicated concentrations of cisplatin (CDDP), either in complete (full) DMEM (black circles) or in the same medium containing 75 µM lysine (**A**), 125 µM arginine (**B**), or 150 µM leucine (**C**) (grey squares). The lysine, arginine, and leucine concentrations in DMEM are 798, 398, and 801 µM, respectively. Cell number was counted after 72 h, and the data are plotted as percent inhibition compared to untreated control cells in full medium. The cisplatin concentration that yielded 50% inhibition is shown as the IC_50_. Each symbol is the mean of three independent experiments performed in duplicate, with error bars representing the standard deviation of the three experiments. Amino acid deprivation caused an upward shift of the inhibition curve, indicating a greater degree of inhibition. (**D**,**E**) Dih10 cells (**D**) and IGROV-1 cells (**E**) were plated at a low density, and cultured in either complete medium (control), medium containing 20 µM lysine (low Lys), complete medium containing 3 µM cisplatin (CDDP), or medium containing 20 µM lysine and 3 µM cisplatin (low Lys/CDDP). Two weeks later, they were fixed, stained, and photographed. The numbers below the wells are the optical absorption after dissolving the stain in acid, and are the mean ± SD of three independent experiments derived from duplicate wells. (**F**–**I**) Dih10 cells were cultured for 6 h in complete DMEM (control) in the presence of 100 µM cisplatin (**G**,**I**) or the indicated cisplatin concentration (**F**,**H**). As noted, some cells were cultured in lysine-deficient medium (Lys-). (**F**,**G**) Cells were extracted, and proteins were analyzed by SDS-PAGE/immunoblotting using antibodies against actin and cleaved caspase-3. (**F**) Protein bands were scanned on a Li-Cor Odyssey instrument, and the sum of the density of the 17 and 19 kDa cleaved caspase-3 bands was normalized to the respective actin bands. Each symbol is from an independent experiment, with the bar height representing the mean value of all experiments and the error bars indicating the standard deviation. (**G**) A full blot is shown. (**H**) The medium and cells were harvested separately, and lactate dehydrogenase (LDH) activity in the medium and cell extracts was measured in a coupled spectrophotometric assay. Data are presented as the percent of LDH activity in the medium. (**I**) Cells were stained using an anti-ɣ-H2A.X antibody and a Texas Red-conjugated secondary antibody, and nuclei were counterstained with DAPI. The scale bar is 50 µm and magnification was 20 × . The number of ɣ-H2A.X-positive foci per cell were counted in 50 cells per condition in four independent experiments, yielding a total of 200 cells counted per condition. The numbers shown are the mean ± SD of the number of foci per cell. *, **, ***, and **** indicate *p* < 0.05, < 0.01, < 0.001, and < 0.0001, respectively, for the indicated comparisons or, in Panels D and E, for comparison to the control. # and ## in Panels D and E indicate *p* < 0.05 and < 0.01, respectively, for comparison to the individual conditions of cisplatin treatment or lysine deprivation. CDDP, cisplatin.

Since cisplatin’s biochemical effects were augmented by amino acid deprivation, we assessed whether cisplatin’s cytotoxic effects were also amplified by amino acid deprivation. Culturing Dih10 cells for 72 h in medium containing limiting concentrations of lysine, arginine, or leucine increased the cells’ sensitivity to the growth inhibitory effects of 0.1–10 µM cisplatin, reducing the IC_50_ in each case by ~ 50% (Fig. 3A–C). The concentrations of lysine, arginine, and leucine were 75, 125, and 150 µM, respectively, compared to 798, 398, and 801 µM in full medium, respectively. Consistent with these data, cisplatin’s inhibition of clonal cell growth was augmented by limiting the lysine concentration, both in Dih10 cells and the ovarian cancer cells (Fig. 3D,E).

Cisplatin cytotoxicity is mediated primarily via apoptosis^1^, but the combination of cisplatin and amino acid deprivation could potentially cause cellular necrosis. Over the 6 h time frame of the biochemical experiments presented in Figs. 1 and 2, Dih10 cells showed no significant increase in apoptosis at 10, 25, 50, or 100 µM cisplatin, as measured by caspase 3 activation (Fig. 3F,G; the blot in Fig. 3G shows 100 µM cisplatin). Similarly, 6 h of lysine deprivation did not increase apoptosis of Dih10 cells (Fig. 3F,G). However, the combination of 50 and 100 µM cisplatin with lysine deprivation significantly increased apoptosis (Fig. 3F,G). The increased apoptosis under the condition of 50 and 100 µM cisplatin combined with lysine starvation correlates with the profound decrease in PRPP that occurred under these conditions (Fig. 1A). We found no increase in cell necrosis as measured by lactate dehydrogenase loss into the medium at 10, 25, 50, or 100 µM cisplatin alone or when combined with lysine deprivation (Fig. 3H). We conclude that amino acid deprivation augments cisplatin-induced apoptosis, but does not induce cellular necrosis.

DNA strand breaks are characteristic of cisplatin treatment, and 100 µM cisplatin increased the number of double-stranded DNA breaks in Dih10 cells more than 16-fold [Fig. 3I; DNA breaks were measured by counting γ-H2AX-positive foci^23^]. Lysine deprivation increased DNA breaks 3.8-fold, which was not significant compared to control cells, but combining lysine deprivation with cisplatin increased DNA breaks 35-fold (Fig. 3I).

### Cisplatin and intermittent amino acid deprivation decrease tumor growth in mice

**Figure 4:**
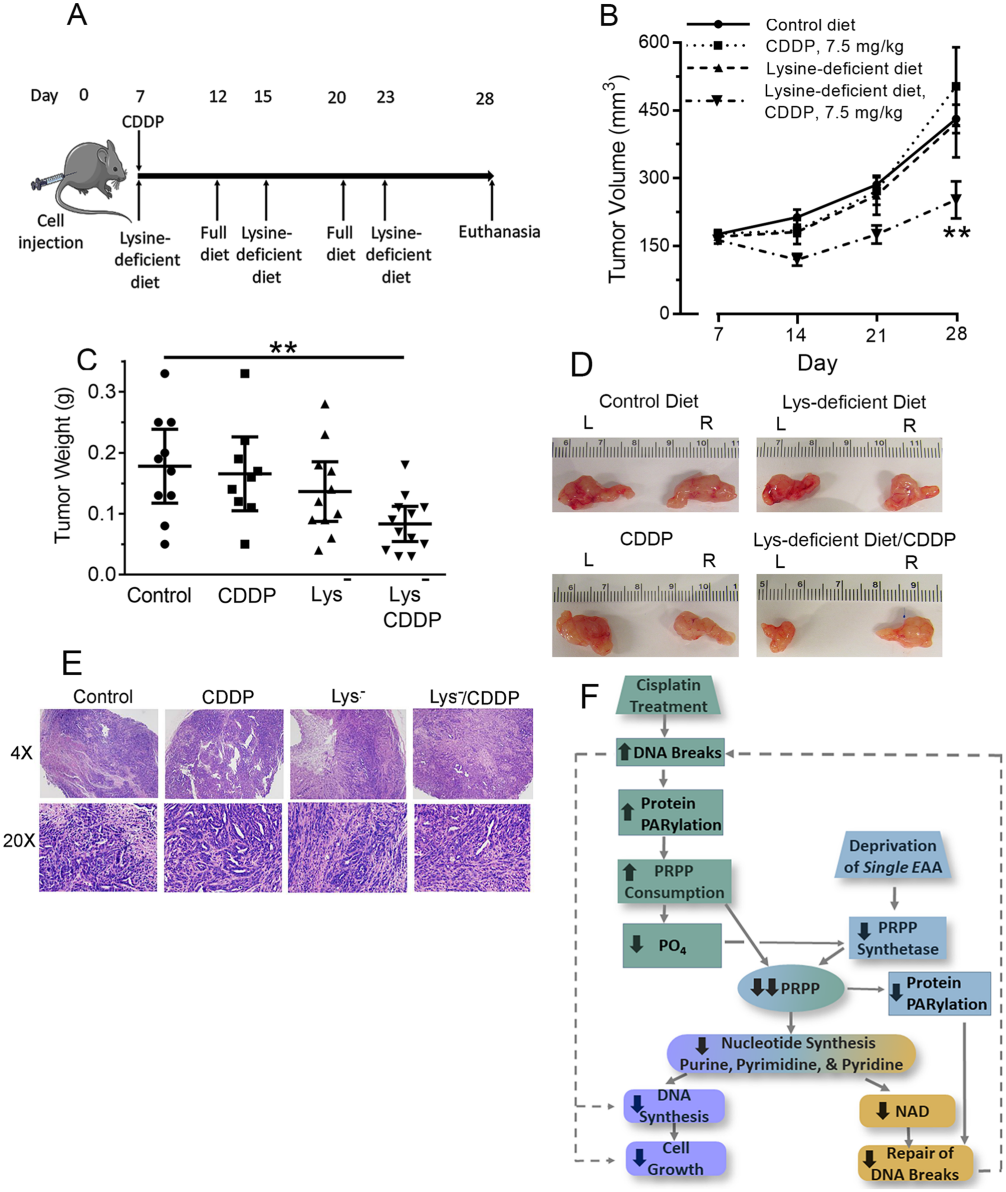
Cisplatin and intermittent amino acid deprivation decrease tumor growth in mice. (**A**) Nude (*Nu*/*Nu*) mice were injected on day zero with 1.7 × 10^6^ Dih10 cells subcutaneously into both flanks. On day 7, the mice were randomly assigned to four groups: Group 1, fully-supplemented diet throughout the study; Group 2, fully-supplemented diet throughout the study with one injection of 7.5 mg/kg cisplatin; Group 3, three 5 d periods of a lysine-free diet separated by two 3 d periods of fully-supplemented diet; Group 4, cisplatin injection followed by the cyclical lysine-free/fully supplemented diet. (**B**) Tumor volumes were measured weekly using a digital caliper. Group 1 mice, solid line, n = 5; Group 2 mice, dotted line, n = 5; Group 3 mice, dashed line, n = 6; Group 4 mice, line with alternating dots and dashes, n = 6. All mice had bilateral tumors, except for one mouse each in Groups 2 and 3, which had only one tumor. Thus, each symbol represents the mean of 10 tumors in Group 1, nine tumors in Group 2, 11 tumors in Group 3, and 12 tumors in Group 4; error bars are the standard deviation. (**C**,**D**) At the time of euthanasia on day 28, tumors were removed, weighed (**C**), and photographed (**D**). Each symbol in Panel C represents one tumor; the wide horizontal line is the mean value, and the error bars are the standard deviation. The photographs in Panel D show tumors from the left (L) and right (R) flank of the same mouse for each of the four conditions. In Panels B and C, ** indicates *p* < 0.01. (**E**) Hematoxylin and eosin stains of representative tumors from each of the four groups of mice. The samples are shown under 4 and 20 × magnification. All four samples showed some fibrosis with prominent spindle cells. (**F**) Cisplatin induces DNA breaks, which increase protein PARylation and PRPP consumption, leading to a decrease in intracellular phosphate. Depriving cells of an essential amino acid decreases PRPP synthetase activity, and when combined with a decrease in intracellular phosphate, further decreases enzyme activity. The decrease in PRPP synthetase activity and increase in PRPP consumption markedly reduces PRPP availability, thereby reducing purine nucleotide synthesis and retarding protein PARylation and repair of DNA breaks; although not studied here, the decrease in PRPP likely also decreases pyrimidine and pyridine nucleotide synthesis. Ultimately, the decrease in PRPP decreases DNA synthesis and intracellular NAD, leading to reduced cell growth and repair of DNA strand breaks. Dashed lines show positive feed-forward and feed-back loops. *CDDP* cisplatin; *EAA* essential amino acid; *PRPP* phosphoribosyl-pyrophosphate.

The increased cytotoxicity observed by combining amino acid deprivation with cisplatin suggested that the anti-tumor effects of cisplatin could be augmented by amino acid deprivation in vivo. We chose to use a nude (*Nu/Nu*) mouse allograft tumor model with intermittent deprivation of lysine. Seven days after injecting Dih10 cells into the flanks of the mice, we randomized them into four different groups: Group 1, fully supplemented diet; Group 2, fully supplemented diet with one injection of 7.5 mg/kg cisplatin; Group 3, three 5 d periods of a lysine-free diet separated by two 3 d periods of a fully-supplemented diet; and Group 4, the combination of cisplatin and the cyclical lysine-free/fully-supplemented diet (Fig. 4A). We euthanized the mice at 28 d.

We chose a moderately-high cisplatin dose that does not significantly reduce weight in nude mice^24^, and, from pilot studies, a lysine deprivation protocol that reduced the serum lysine concentration by about 50%, but resulted in less than 12% weight loss. The average weight change in each mouse group was: 2.7% increase in Group 1; 1.3% decrease in Group 2; 10.3% decrease in Group 3, and 11.6% decrease in Group 4. In animals in Groups 3 and 4, the average serum lysine concentration was reduced by ~ 55%, with no change in the serum concentration of other amino acids (Table 2). Mice in all four groups appeared completely normal in behavior and activity throughout the study.

**Table 2:** Mouse serum amino acid concentrations.

Amino acid	Control	Lys^−^	CDDP	Lys^−^/CDDP
	µmol/L			
Ala	382	355	382	522
Arg	115	108	117	117
Asn	36	33	32	39
Asp	22	21	19	17
Cys	5.5	3.9	4.8	4.0
Gln	505	464	442	447
Glu	72	56	61	51
Gly	391	415	393	636
His	81	95	91	92
Ile	80	92	98	86
Leu	129	152	147	130
Lys	296	141***	304	129***
Met	57	54	83	96
Phe	64	71	74	70
Pro	40	44	41	51
Ser	162	192	173	220
Thr	216	209	217	278
Trp	40	34	34	34
Tyr	68	47	64	56
Val	153	185	189	192

Mice were injected in the flank with Dih10 hepatoma cells, and received either normal rodent chow (Control) or three cycles of lysine-deficient chow (Lys^-^) for 5 days followed by 3 days of normal chow as depicted in Fig. 4A. Some animals on either normal chow or lysine-deficient chow received one injection of cisplatin (CDDP). At the end of the third cycle of the lysine-deficient diet, the animals were euthanized, and blood was obtained by cardiac puncture. Amino acids in the serum were measured by high performance liquid chromatography coupled to post-column derivitization with ninhydrin. Data are mean values from five animals in each group; *** = p < 0.001 compared to control animals.

We measured tumor volume weekly and tumor weight at the time of euthanasia. Neither cisplatin alone nor lysine deprivation alone had an effect on tumor volume (Fig. 4B). The combination of cisplatin and amino acid deprivation reduced tumor volume at all three time points, with a significant 50% reduction in volume compared to Group 1 mice at 28 d (Fig. 4B). Consistent with the tumor volumes, cisplatin or lysine deprivation alone had no significant effect on tumor weight measured at 28 d, but the combination of the two markedly decreased mean tumor weight to 45% of the weight in control mice fed a fully-supplemented diet (Fig. 4C). The decrease in tumor weight by the combination of cisplatin and lysine deprivation was reflected in a visible decrease in tumor size (Fig. 4D). Tumor histopathology was unchanged by cisplatin treatment, lysine deprivation, or the combination of the two modalities (Fig. 4E).

## Discussion

We found that cisplatin and deprivation for a single essential amino acid individually reduced cellular availability of PRPP. When combined together, the two modalities profoundly and synergistically decreased PRPP, decreased tumor cell growth in vitro and in vivo, and decreased DNA synthesis, while increasing cellular apoptosis and DNA strand breaks (Fig. 4F).

The decrease in PRPP in cisplatin-treated cells was in part from increased PRPP consumption due to increased protein PARylation (Fig. 4F), because PARP1 knock-down using an shRNA returned PRPP availability in cisplatin-treated cells to values near those found in control cells. We also tried a pharmacological approach to knock down PARP activity, but three different PARP inhibitors—olaparib, rucaparib, and E7449—unexpectedly decreased PRPP availability at concentrations that inhibit PARP, possibly through off-target effects. The PARP1 shRNA did not fully restore PRPP to control values in cisplatin-treated cells, likely because: (1) PARP1 knock-down was incomplete as shown in Fig. 2J; (2) other PARPs could be present in Dih10 cells; and (3) cisplatin decreased intracellular phosphate, thereby decreasing PRPP synthetase activity and PRPP production (discussed in the next paragraph). The PARP1 shRNA increased PRPP availability under all conditions tested, including in control cells, suggesting that a substantial amount of PRPP is consumed by protein PARylation, even under normal, non-stressed conditions. In lysine-starved, cisplatin-treated cells, the PARP1 shRNA had only a modest effect on PRPP availability, and this is likely due to low PRPP synthetase activity. Overall, these data demonstrate a dynamic balance between the activities of PRPP synthetase and PARP1 in determining PRPP availability.

We did not expect cisplatin would reduce intracellular phosphate, but each ADP-ribose unit in poly(ADP-ribose) contains two phosphate moieties, and multiple proteins undergo PARylation after cisplatin generation of DNA adducts and strand breaks^25^. Since each poly(ADP-ribose) chain can contain up to 250 ADP-ribose units and each protein can have several chains attached, it seems possible that cisplatin could transiently decrease the intracellular phosphate concentration via increased PARP activity^25^. At the normal intracellular concentration of 1–2 mM inorganic phosphate, even a modest decrease in phosphate will profoundly reduce PRPP synthetase activity, due to a steep slope in the phosphate response curve of the enzyme between 0 and 10 mM phosphate^22^.

The half-lives of the two main PRPP synthetases have not been reported previously, due in part to a lack of specific antibodies. Although depriving cells of lysine does not immediately nor fully stop protein synthesis^4^, we found that PRPP synthetase activity was decreased by 50% after ~ 8 h of lysine deprivation. This measurement almost certainly overestimates the enzymes’ true half-lives, but it indicates that PRPP synthetase activity would be reduced by about 40% during the 6 h time frame of most of our in vitro experiments. The 40% reduction in enzyme activity by amino acid deprivation combined with a 16% reduction in inorganic phosphate by cisplatin would be expected to reduce cellular PRPP synthetase activity, and thus PRPP production. Reduced PRPP synthetase activity during amino acid deprivation is yet another mechanism, in addition to the reduction in transketolase activity that we found previously^6^, of reduced PRPP production in cells deprived of an essential amino acid.

Reduced PRPP availability likely led to the decrease in purine and DNA synthesis and increase in DNA strand breaks in cells treated with cisplatin and deprived of an amino acid. PRPP is a rate-limiting substrate for de novo purine synthesis and even small reductions in PRPP availability reduce purine synthesis^13^. Reduced purine synthesis, in turn, likely contributed to reduced DNA synthesis, but the latter occurred at a time when the intracellular ATP concentration was unchanged. This suggests that different pools of purine nucleotides may exist, and that DNA synthesis draws from a pool of newly-synthesized nucleotides. We previously observed reduced DNA synthesis during amino acid deprivation in the absence of a change in the intracellular concentration of any purine nucleotide^4^.

Lysine deprivation prevented the increase in protein PARylation that occurs in cisplatin-treated cells. The inhibition of protein PARylation likely explains the marked increase in DNA strand breaks in cisplatin-treated, lysine-deprived cells, and suggests the breaks were from decreased DNA repair, rather than from an increase in the number of breaks (Fig. 4F). The block of protein PARylation by lysine deprivation occurred in the absence of a significant change in total cellular NAD^+^ or PARP1 protein. This suggests, as for DNA synthesis, that protein PARylation may draw from a pool of newly-synthesized NAD^+^ that was contracted due to the striking reduction in PRPP in cisplatin-treated, lysine-deprived cells.

Consistent with our results, previous workers have found that PRPP synthetase is highly expressed in cisplatin-resistant human breast cancer cells and that silencing the enzyme reverses cisplatin resistance^26^. Moreover, knockdown of transketolase-like 1, which functions similarly to transketolase, increases cisplatin cytotoxicity in nasopharyngeal carcinoma cells by reducing ribose 5-phosphate, and therefore PRPP^27^.

We specifically chose lysine as the main essential amino acid to be restricted both in the cell and animal studies, because of its limited intracellular role, other than being required for protein synthesis. Lysine can be a precursor to carnitine, via trimethyllysine; however, carnitine is present in fetal bovine serum and most animals receive sufficient carnitine in their diets and do not synthesize it from lysine. Thus, the effects of lysine starvation we observed, both in vitro and in vivo, are likely from reduced lysyl t-RNA charging and protein synthesis. Arginine and leucine, the two other amino acids we restricted, are involved in other biochemical reactions—including sensing of amino acid availability by mTORC1^28^. Since they had similar effects as lysine restriction, it appears that at least in regulating PRPP availability, they likely acted through reduced protein synthesis.

Mice on the lysine-deficient diet lost about 11% of their body weight. Although an 11% weight loss would be hazardous for a non-obese human, the metabolic rate of a 30 g mouse is seven times greater than that of a 70 kg human^29^. Thus, a nutritionally-deficient diet will have a much smaller effect on a human than a mouse, and multiple studies in cancer patients have shown that depriving patients of the essential amino acid methionine for recurring cycles of 3 to 7 days does not lead to weight loss or other adverse effects^30–35^. Similarly, recurrent cycles of arginine deiminase or arginase, both of which markedly lower serum arginine concentrations, are well tolerated by cancer patients^36–41^.

Combining amino acid deprivation with cisplatin could allow the drug to be used in tumors that show resistance to the drug. Thus, we found a reduction in tumor size when lysine deprivation was added to cisplatin at the highest cisplatin dose tolerated by nude mice, which by itself had no effect^24^. This is consistent with hepatomas being largely unresponsive to most standard chemotherapeutic agents, including cisplatin^42^.

Our study has several limitations. First, most of the work was done in mouse hepatoma cells. Since the two main PRPP synthetases are ubiquitously expressed, the results should be applicable to other cell types, but confirmatory experimentation is needed. Second, in several assays we followed the incorporation of radioactively-labeled compounds into labeled products. Such assays are highly sensitive, but they rely on equal cellular uptake of the labeled compounds and no change in their intracellular pools under the conditions tested. Third, we focused on changes in PRPP and PRPP synthetase activity. Other metabolites or enzymes could have changed during the 6 h of lysine deprivation in the cultured cell experiments, but identifying such changes and whether they augmented cisplatin efficacy was beyond the scope of this work. And fourth, we used only male mice in the in vivo studies; while seemingly unlikely, it is possible that female mice could have responded differently.

In conclusion, combining cisplatin with brief cycles of deprivation for an essential amino acid could be beneficial in cancer treatment, particularly in cisplatin-resistant tumors. The potential of nutrient limitation in cancer therapy is exemplified by combining chemotherapeutic agents with L-asparginase— which lowers serum asparagine concentrations—as a standard approach to treat childhood acute lymphoblastic leukemia, but asparagine auxotrophy is limited to a subset of leukemias^43^.

## Methods

### Materials

Reagents were of the highest grade commercially available; the source of antibodies and key reagents is shown in Table 3. Cisplatin was made fresh just before use by dissolving solid cisplatin in aqueous 0.9% sodium chloride.

**Table 3:** Key resources.

Product name	Company	Cat #
**Antibodies**		
β-actin	Santa Cruz	SC-47778
P-Histone H2A.X	Cell Signaling	2577
Cleaved caspase-3	Cell Signaling	9661
PARP1	Cell Signaling	9532
PAR polymers	Trevigen	4335-MC-100-AC
**Amino acid-deficient culture medium**		
DME high glucose minus arg, leu, lys, met	Athenaes	0420
DME low glucose minus arg, gln, leu, lys	Sigma	D9443
RPMI minus arg, lys	Thermo-Fisher	89984
**Radioactive chemicals**		
[8-^14^C]Hypoxanthine	Moravek, Inc	MC-147
[1-^14^C]D-Glucose	Moravek, Inc	MC228W
[^14^C]Formic acid sodium salt	Moravek, Inc	MC173
[Methyl-^3^H]Thymidine	Moravek, Inc	MT6039
**Reagents**		
Cell Titer-Glo 2.0 Assay	Promega	G9248
Cis-Diammineplatinum(II) dichloride	Millipore-Sigma	P-4394
Human epidermal growth factor	Millipore-Sigma	E9644
Lipofectamine 2000	Thermo-Fisher	11668019
5-Phospho-D-ribose 1-diphosphate, pentasodium salt	Millipore-Sigma	P-8296
D-Ribose 5-phosphate, disodium salt	Millipore-Sigma	83875
Thiazolyl blue tetrazolium bromide	Millipore-Sigma	M2128
**Rodent chow**		
Amino acid-defined diet	Teklad	86529
Amino acid-defined diet minus lysine	Teklad	99386

Reagents were of the highest grade commercially available. Cisplatin was made fresh just before use by dissolving it in an aqueous solution of 0.9% sodium chloride.

### Cell origin and culture

Dih10 cells were derived previously from a diethylnitrosamine-induced mouse hepatoma, and were confirmed as hepatic in origin, since they stained positive for alpha fetoprotein and produced albumin^44^. They were grown in DMEM containing 4.5 g/L glucose, supplemented with 20% fetal bovine serum, 10 mg/L insulin, 10 mg/L hydrocortisone, 20 µg/L epidermal growth factor, and 1 mM phenobarbital^44^. The IGROV-I ovarian cancer cell line was established in France in 1984 from a patient with Stage III ovarian cancer, and was received as a gift; it stained positive for PAX8, a marker of Mullerian differentiation^45^. The cells were grown in RPMI-1640 medium supplemented with 10% fetal bovine serum. Both cell lines were used between passages three to ten, and were negative for *Mycoplasma* infection.

### General experimental conditions

Cells were seeded the evening before an experiment in their normal growth medium at a density corresponding to 350,000 cells per well of a 12 well plate, unless stated otherwise. On the day of the experiment, the cells were washed once with phosphate-buffered saline (PBS), and incubated for the indicated times in 0.5 ml experimental medium per well of a 12 well plate (correspondingly lower and higher volumes for smaller and larger wells, respectively). This medium was made fresh the day of the experiment, and consisted of either DMEM containing 4.5 g/L glucose (Dih10 cells) or RPMI-1640 medium (IGROV-I cells). The DME medium lacked arginine, leucine, lysine, and methionine; the RPMI medium lacked arginine and lysine. To generate full medium, the missing amino acids were added to their normal concentrations of 398 µM arginine, 801 µM leucine, 798 µM lysine, and 200 µM methionine for DME, and 948 µM arginine and 219 µM lysine for RPMI. To generate medium deficient in one amino acid, the other amino acids were added to their normal concentration, and the deficient one was either not added to make medium completely deficient in that amino acid, or added to the indicated concentration. In some experiments, lysine-deficient DMEM contained glucose at 1 g/L, and either lacked sodium bicarbonate or contained 15 mM NaHCO_3_; in the latter cases, 20 mM Hepes, pH, 7.4 was added to the medium. All experimental media were supplemented with 10% fetal bovine serum that had been dialyzed against 0.9% sodium chloride.

### Measurement of PRPP availability, and purine and DNA synthesis

Cells were incubated in 12 well (PRPP availability and DNA synthesis) or 6 well plates (purine synthesis) for the indicated times.

For PRPP availability, [8-^14^C]-hypoxanthine (1 µCi, 58 mCi/mmol) was added during the last 20 min of incubation, and the cells were washed twice with PBS, extracted in 1 mM NaOH, and the extracts spotted onto squares of diethylaminoethyl cellulose paper^6^. The paper squares were washed three times with 1 mM ammonium formate, dried, and radioactivity on the squares was measured.

For purine synthesis, [^14^C]-formate (10 µCi, 56 mCi/mmol) was added during the last 90 min of incubation, and the cells were extracted in 0.4 N perchloric acid^4, 6^. The extracts were boiled for 70 min to reduce all purine nucleotides and nucleosides to purine bases, and supernatants were applied to Dowex 50 columns eluted with 6 N HCl.

For DNA synthesis, [^3^H]-thymidine (1 µCi, 70 Ci/mmol) was added during the last 60 min of incubation, and the cells were washed once with PBS and extracted with 10% trichloroacetic acid^4, 6^. The resulting precipitates were collected on glass fiber discs, which were washed with trichloroacetic acid, dried, and transferred to scintillation vials.

In all cases, radioactivity was measured by liquid scintillation counting using 2,5-diphenyloxazole and p-bis-[2-(5-phenyloxazolyl)]-benzene as primary and secondary fluorophores, respectively.

### Measurement of ATP

Cells were incubated in 96 well plates, and at the end of the incubation period, ATP was measured against a standard curve using the Cell Titer- Glo 2.0 kit (Promega) following the manufacturer’s instructions.

### Measurement of NAD

Cells in 12 well plates were extracted in perchloric acid, and NAD^+^ in the neutralized extracts was measured in an enzyme-cycling colorimetric assay following thiazolyl blue tetrazolium bromide reduction^46^.

### Measurement of glucose oxidation via the oxidative pentose phosphate pathway and incorporation into DNA and RNA via the non-oxidative pentose phosphate pathway

To measure glucose oxidation via the oxidative pentose phosphate pathway, cells were incubated in T25 flasks in bicarbonate-deficient DMEM containing 1 g/L glucose and 20 mM Hepes^6, 7^.[1-^14^C]-glucose (1 µCi, 58 mCi/mmol) was added during the last 60 min of incubation, and the flasks were immediately capped with rubber stoppers that had an attached center well containing fluted filter paper. At the end of the incubation period, perchloric acid was injected into the medium to a final concentration of 0.4 N, and 10 mM NaOH was injected into the center well. The perchloric acid simultaneously extracts the cells and converts HCO_3_^−^ to CO_2_; the CO_2_ was collected in the wells by conversion back to HCO_3_^−^ by incubating the flasks overnight. The collected radioactive CO_2_ was quantified by transferring the wells to scintillation vials and measuring radioactivity by liquid scintillation counting.

To measure glucose incorporation into DNA and RNA via the non-oxidative pentose phosphate pathway, cells were incubated in 12 well plates in DMEM containing 1 g/L glucose, with [1-^14^C]-glucose (1 µCi, 58 mCi/mmol) added during the last 60 min of incubation^6, 18^. The cells were washed once at the end of the incubation period with PBS and extracted in 10% trichloroacetic acid. The extracts were washed with trichloroacetic acid and heated at 80 °C for 30 min to solubilize precipitated DNA and RNA; the resulting supernatants were transferred to scintillation vials, and radioactivity was measured.

### Measurement of HPRT and PRPP synthetase activity

Cells in six well plates were washed with ice-cold Tris-buffered saline, and extracted either by repeated passage through a 27 g needle (HPRT) or by three freeze–thaw cycles (PRPP synthetase). The extracts were clarified by centrifugation, and incubated with [8-^14^C]hypoxanthine (58 mCi/mmol, final concentration 20 µM) in the presence or absence of 0.4 mM PRPP (HPRT), or with 0.7 mM ATP and [8-^14^C]adenine (54 mCi/mmol, final concentration 30 µM) in the presence or absence of 0.5 mM ribose 5-phosphate (PRPP synthetase)^5, 13^. Sufficient adenine phosphoribosyl-pyrophosphate transferase activity was present in the extracts that exogenous enzyme was not necessary in the PRPP synthetase assay. The samples were then processed as for measuring PRPP availability. In the PRPP synthetase assay, sodium phosphate, pH 7.4 was added to the final concentration indicated. Enzyme activity was calculated as the difference between the presence and absence of PRPP or ribose 5-phosphate for HPRT or PRPP synthetase, respectively, and was linear with time and protein concentration.

### Measurement of inorganic phosphate

Cells in six well plates were washed twice with Tris-buffered saline and extracted in 6% sodium dodecyl sulfate. The extracts were sonicated, and phosphate was measured in an improved ammonium molybdate-malachite green assay^47^.

### shRNA knockdown of PARP1

A PARP1 shRNA plasmid containing the sequence CCTTGGAAACATGTATGAACTC (SHCLNG-NM_007415) and a non-target shRNA control plasmid (SHC016) were purchased from Millipore-Sigma. They were co-transfected into 293 T cells with pCMV-Δ8.2R and pCMV-VSV-G expression vectors using Lipofectamine 2000. Medium supernatant containing assembled lentivirus was harvested 48 h later, filtered, and added twice, 24 h apart, to Dih10 cells. Twenty-four hours after the second addition, the Dih10 cells were placed in 1 µg/ml puromycin, and stable clones were selected by limiting dilution. PARP protein was assessed by Western blotting and normalized to actin. Experiments were done with cells obtained from three independent infections.

### Immunoblotting

Cells were incubated in 12 well plates for the indicated times, and extracted in situ in sodium dodecyl sulfate sample buffer^6^. Proteins were separated by SDS-PAGE, transferred to a nitrocellulose membrane, and the membrane was incubated with the indicated primary antibody, followed by a horseradish peroxidase-conjugated secondary antibody. Proteins were quantified by chemiluminescence using a Li-COR Odyssey Scanner and Image Studio Version 5.2 software (https://www.licor.com/bio/image-studio-lite/download).

### Assessment of cell growth

Cells were seeded at 30,000 cells per well in 24 well plates, and cultured under the indicated conditions. After 72 h, cells from triplicate wells were counted twice using a hemocytometer.

### Clonogenic assay

Cells were seeded at 300 cells per well in six-well plates and cultured under the indicated conditions. After 2 weeks of culture, they were fixed in methanol, stained with crystal violet, and photographed. Stained cells were quantified by dissolving the crystal violet in acetic acid, and measuring optical density at 590 nm.

### Measurement of lactate dehydrogenase (LDH) activity

Cells in a 96 well plate were placed in 0.2 ml of experimental DMEM medium containing 10% dialyzed heat-inactivated (60 °C for 30 min) fetal bovine serum. After 6 h of incubation, the media was harvested and the cells were extracted in situ in 0.9% Triton X-100. LDH activity in the media and cell extracts was measured separately in a coupled reaction following (1) lactate oxidation to pyruvate, with consequent NAD reduction to NADH, and (2) NADH reduction of iodonitrotetrazolium chloride to a colored formazan product measured at 490 nm^48^. The assay was linear with time and cellular protein concentration, and results are presented as the percent of total LDH activity in the medium.

### Immunofluorescence staining

Cells plated on glass slide cover slips (10,000 cells per slip) were incubated for the indicated times, fixed with 3.7% formaldehyde in PBS, permeabilized with 0.5% Triton X-100, and blocked with 2% bovine serum albumin. They were stained with phosphohistone H2A.X antibody followed by a Texas red-conjugated secondary antibody. Nuclei were counter-stained with 4′,6-diamidino-2-phenylindole (DAPI).

### Mouse xenograft model

Nude (*Nu*/*Nu*) male mice 8–12 weeks old and weighing an average of 28.2 g were housed four animals per cage in a pathogen-free environment with a 12 h light–dark cycle. At the start of the experiment, they were anesthetized briefly with 1.5% isoflurane, and injected subcutaneously in both flanks with 1.7 × 10^6^ Dih10 cells mixed 1:1 with Matrigel. For 7 d they received fully supplemented rodent chow (Teklad Amino Acid-Defined Diet), at which time they were randomly divided into four groups, assuring that the average tumor volume was similar in each group. Group 1 animals received the fully supplemented chow for the remainder of the study. Group 2 animals received fully supplemented chow and a single intraperitoneal injection on day seven of 7.5 mg/kg cisplatin in 0.9% NaCl, ~ 280 µl per mouse. Group 3 animals received the following diet: three 5 d periods of lysine-free chow (same formulation as fully supplemented chow but lacking lysine, Teklad Custom Diet Minus Lysine) separated by two 3 d periods of fully supplemented chow (Fig. 4A). Group 4 animals received the same 7.5 mg/kg cisplatin injection as Group 2 mice followed by the cyclical lysine-free/fully-supplemented chow diet as Group 3 mice.

Tumor size was measured weekly by digital caliper on non-anesthetized animals in a blinded fashion, and tumor volume was calculated by using the following formula: tumor volume = 0.5236 (tumor length × tumor width^2^)^49^. All mice were euthanized on day 28 by cervical dislocation after being anesthetized with 2% isoflurane; tumors were removed, weighed, and photographed. A pilot experiment indicated that to observe at least a 50% difference between Group 1 and 4 mice (80% power, α = 0.05) required five mice in each group. We started with six mice per group, but excluded one mouse each from Groups 1 and 2, because no palpable tumors had developed by day 7. Thus, the study consisted of five mice in Groups 1 and 2, and six mice in Groups 3 and 4. Primary outcomes were tumor size and weight. The studies were conducted at the University of California, San Diego, and were approved under Protocol #S11208 by the UCSD Institutional Animal Care and Use Committee, Association for Assessment and Accreditation of Laboratory Animal Care (AAALAC) #000503. The studies were conducted according to the National Academies of Sciences, Engineering, and Medicine Institute for Laboratory Animal Research (ILAR) Guide to the Care and Use of Laboratory Animals.

### Measurement of serum amino acids

Blood obtained by cardiac puncture at the time of euthanasia was allowed to clot, and the resulting serum was extracted using 2% 5-sulfosalicylic acid. Amino acids in the serum were analyzed on a Biochrom 30 amino acid analyzer using a PEEK (polyether-ketone-ketone) lithium ion exchange column, with ninhydrin post-column derivatization. They were quantified by comparison to authentic standards using calibration curves with R^2^ > 0.99.

### Data analysis

Symbols in bar graphs represent the mean of duplicate samples, with each symbol in a given condition derived from an independent experiment. Comparisons among conditions were made by a one-way ANOVA, except in Fig. 2K where a two-way ANOVA was used. In all cases, Sidak’s multiple comparisons post-test analysis was used in GraphPad Prism 7.0. A *p* value of < 0.05 was considered significant; the absence of a comparison between two conditions means the difference was not significant. Further statistical details are in the figure legends.

To determine if combining cisplatin with amino acid deprivation yielded an additive, synergistic, or antagonistic effect, we considered cisplatin as one drug and amino acid deprivation as a second drug. We then calculated the coefficient of drug interaction (CDI) using the formula CDI = AB/A × B, where A, B, and AB are the ratios of the results with cisplatin alone, amino acid deprivation alone, and the combination of cisplatin and amino acid deprivation, respectively, over the result in control untreated cells in full medium^50, 51^. CDI > 1 indicates antagonism, CDI = 1 indicates an additive effect, and CDI < 1 indicates synergy, with the lower the value, the greater the synergy.

## Data Availability

The data of this work are all within the manuscript.

## Abbreviations

CDDP: Cisplatin
CDI: Coefficient of drug interaction
HPRT: Hypoxanthine phosphoribosyltransferase
NAD^+^: Nicotinamide adenine dinucleotide
PARP: Poly(ADP-ribose) polymerase
PRPP: 5-Phosphoribose-1-pyrophosphate
PBS: Phosphate-buffered saline
